# 3,3-Dimethyl-1,2,3,4-tetra­hydro­cyclo­penta­[*b*]indole-1,2-dione (bruceolline E)

**DOI:** 10.1107/S1600536812000517

**Published:** 2012-01-11

**Authors:** Jason A. Jordon, Jeanese C. Badenock, Gordon W. Gribble, Jerry P. Jasinski, James A. Golen

**Affiliations:** a Department of Biological and Chemical Sciences, University of the West Indies, Cave Hill, Barbados; b Department Chemistry, Dartmouth College, Hanover, NH 03755-3564, USA; c Department of Chemistry, Keene State College, 229 Main Street, Keene, NH 03435-2001, USA

## Abstract

The title compound, C_13_H_11_NO_2_, crystallizes with two mol­ecules in the asymmetric unit. The crystal packing is stabilized by N—H⋯O hydrogen bonds, which link the mol­ecules into chains along [10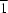], and weak C—H⋯O inter­actions.

## Related literature

For the first isolation of bruceolline E as yellow needles, see: Ouyang *et al.* (1994 ▶). For the first total synthesis of bruceolline E in three steps from the known ethyl indole-1-carboxyl­ate, see: Jordan *et al.* (2011 ▶). For examples of similar tandem acyl­ation/Naza­rov cyclization with pyrroles, see: Song *et al.* (2006 ▶). For examples of Naza­rov cyclizations with indoles, see: Bergman & Venemalm (1992 ▶); Cheng & Cheung (1996 ▶); Ishikura *et al.* (2000 ▶); Miki *et al.* (2001 ▶); Churruca *et al.* (2010 ▶). For examples of α-diketone oxidations using selenium dioxide, see: Gribble *et al.* (1988 ▶); Xu *et al.* (2002 ▶); Belsey *et al.* (2006 ▶). For related cyclo­penta­[*b*]indolone alkaloids and their ana­logues, see: Cheng *et al.* (1991 ▶); Garcia-Pichel & Castenholz (1991 ▶); Garcia-Pichel *et al.* (1992 ▶); Proteau *et al.* (1993 ▶); Ekebergh *et al.* (2011 ▶); Kobayashi *et al.* (1994 ▶); Jacquemard *et al.* (2004 ▶); Ploutno & Carmeli (2001 ▶). For standard bond lengths, see: Allen *et al.* (1987 ▶).
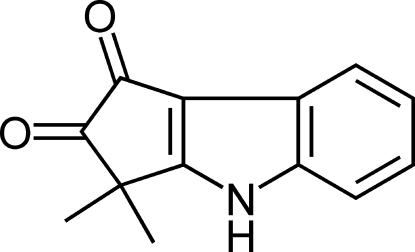


## Experimental

### 

#### Crystal data

C_13_H_11_NO_2_
*M* *_r_* = 213.23
Triclinic, 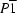

*a* = 9.1091 (7) Å
*b* = 11.5337 (8) Å
*c* = 11.8745 (9) Å
α = 63.230 (7)°
β = 80.596 (6)°
γ = 79.970 (6)°
*V* = 1091.69 (14) Å^3^
*Z* = 4
Mo *K*α radiation
μ = 0.09 mm^−1^
*T* = 170 K
0.28 × 0.25 × 0.24 mm

#### Data collection

Oxford Diffraction Xcalibur Eos Gemini diffractometer
Absorption correction: multi-scan (*CrysAlis RED*; Oxford Diffraction, 2010 ▶) *T*_min_ = 0.976, *T*_max_ = 0.979
10062 measured reflections
5644 independent reflections
4698 reflections with *I* > 2σ(*I*)
*R*_int_ = 0.012

#### Refinement

*R*[*F* ^2^ > 2σ(*F* ^2^)] = 0.053
*wR*(*F* ^2^) = 0.157
*S* = 1.05
5644 reflections
299 parameters
2 restraints
H atoms treated by a mixture of independent and constrained refinement
Δρ_max_ = 0.36 e Å^−3^
Δρ_min_ = −0.20 e Å^−3^

### 

Data collection: *CrysAlis PRO* (Oxford Diffraction, 2010 ▶); cell refinement: *CrysAlis PRO*; data reduction: *CrysAlis RED* (Oxford Diffraction, 2010 ▶); program(s) used to solve structure: *SHELXS97* (Sheldrick, 2008 ▶); program(s) used to refine structure: *SHELXL97* (Sheldrick, 2008 ▶); molecular graphics: *SHELXTL* (Sheldrick, 2008 ▶); software used to prepare material for publication: *SHELXTL*.

## Supplementary Material

Crystal structure: contains datablock(s) global, I. DOI: 10.1107/S1600536812000517/qm2048sup1.cif

Structure factors: contains datablock(s) I. DOI: 10.1107/S1600536812000517/qm2048Isup2.hkl

Supplementary material file. DOI: 10.1107/S1600536812000517/qm2048Isup3.cml

Additional supplementary materials:  crystallographic information; 3D view; checkCIF report

## Figures and Tables

**Table 1:** Hydrogen-bond geometry (Å, °)

*D*—H⋯*A*	*D*—H	H⋯*A*	*D*⋯*A*	*D*—H⋯*A*
N1—H1*NA*⋯O2*A*	0.89 (1)	1.95 (1)	2.8025 (16)	161 (2)
N1*A*—H1*NB*⋯O2^i^	0.92 (1)	1.87 (1)	2.7686 (16)	164 (2)
C9*A*—H9*AA*⋯O1^i^	0.95	2.51	3.376 (2)	152
C12*A*—H12*B*⋯O2^i^	0.98	2.57	3.430 (2)	146

Symmetry code: (i) 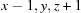.

**Fig. 1.**
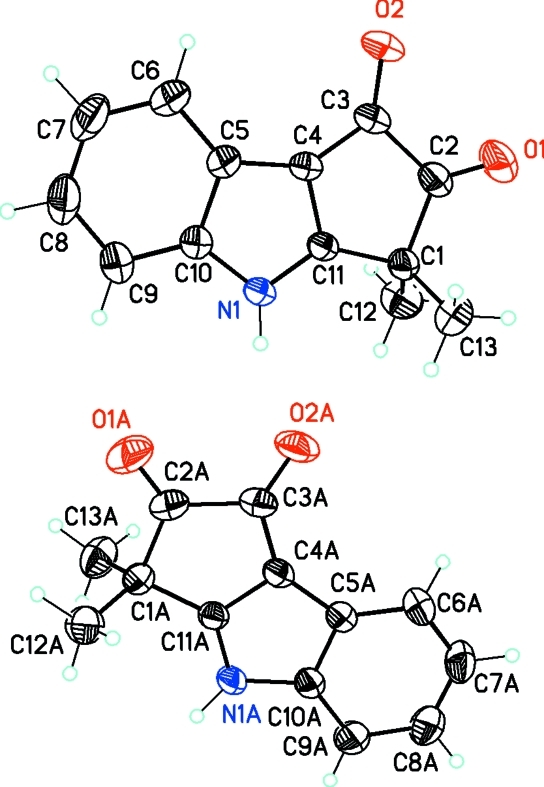
Molecular structure of the title compound with two molecules in the asymmetric unit showing the atom labeling scheme and 50% probability displacement ellipsoids.

**Fig. 2.**
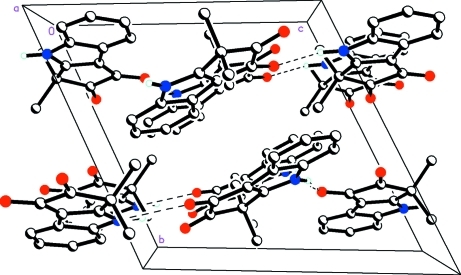
Packing diagram of the title compound viewed along the a axis. Dashed lines indicate N—H···O hydrogen bonds forming infinite 1-D chains along [011]. The remaining H atoms have been removed for clarity.

## supplementary crystallographic information

### Comment

The fused indole alkaloid bruceolline E was first isolated as yellow needles from the root wood *Brucea mollis Wall. Var. tonkinensis Lecomte* and reported by Ohmoto (Ouyang *et al.*, 1994). Our synthesis of this cyclopenta[*b*]indolone natural product included a tandem acylation/Nazarov cyclization sequence (Song *et al.*, 2006) and insertion of the α-diketone functionality using selenium dioxide (Gribble *et al.*, 1988) in 60% yield. Similar Nazarov cyclizations with indoles (Bergman & Venemalm, 1992; Cheng & Cheung, 1996; Ishikura *et al.*, 2000; Miki *et al.*, 2001; Churruca *et al.*, 2010) have been reported. Related α-diketone oxidations using selenium dioxide (Xu *et al.*, 2002; Belsey *et al.*, 2006) and cyclopenta[*b*]indolone natural product alkaloid analogues such as scytonemin (Garcia-Pichel & Castenholz 1991; Garcia-Pichel *et al.* 1992; Proteau *et al.* 1993; Ekebergh *et al.* 2011), nostodione (Kobayashi *et al.*, 1994) and prenostodione (Ploutno & Carmeli, 2001) have also been reported. Our efforts have yielded the first total synthesis of bruceolline E (Jordan *et al.*, 2011), in three steps from the known ethyl indole-1-carboxylate.

We now report herein the first crystal structure of the title compound, C_13_H_11_NO_2_, bruceolline E, which confirms the cyclopenta[*b*] indole moiety and the α-diketone functionalities earlier assigned by NMR methods.

In the crystal structure of the title compound, C_13_H_11_NO_2_, two molecules crystallize in the asymmetric unit (Fig. 1). Bond lengths are in normal ranges (Allen *et al.*, 1987). Crystal packing is stabilized by N—H···O hydrogen bonds (Table 1) and weak C—H···O intermolecular interactions linking these 1-D chains along [011] (Fig. 2).

### Experimental

A solution of 3,3-dimethyl-1,2-dioxo-2,3-dihydro-1H-cyclopenta[*b*] indole-4-carboxylic acid ethyl ester (0.58 g, 2.03 mmol, 1 eq.), anhydrous THF (30 mL) and TBAF (10.2 mL, 1.0 M in THF, 10.2 mmol, 5.02 eq.) was stirred under argon at reflux for 2 h (Jacquemard *et al.*, 2004). The mixture was allowed to cool before quenching with saturated NH_4_Cl. After extraction with CH_2_Cl_2_ (3 x 40 mL), the organic layers were combined, dried over MgSO_4_ and concentrated in vacuo to give a yellow solid. Flash column chromatography (100% EtOAc) gave the product as a yellow solid (0.42 g, 97%). Crystals suitable for X-ray diffraction were grown from methanol [m.p. 562- 563 K (dec); literature value 562- 564 K (dec)].

### Refinement

H1NA and H1NB were located by a Fourier map and refined isotropically. All of the remaining H atoms were placed in their calculated positions and then refined using the riding model with Atom—H lengths of 0.95Å (CH) or 0.98Å (CH_3_). Isotropic displacement parameters for these atoms were set to 1.19-1.20 (CH) or 1.4-1,50 (CH_3_) times *U*_eq_ of the parent atom.

### Crystal data

**Table:**

C_13_H_11_NO_2_	*Z* = 4
*M**_r_* = 213.23	*F*(000) = 448
Triclinic, *P*1	*D*_x_ = 1.297 Mg m^−^^3^
Hall symbol: -P 1	Mo *K*α radiation, λ = 0.71073 Å
*a* = 9.1091 (7) Å	Cell parameters from 5810 reflections
*b* = 11.5337 (8) Å	θ = 3.3–32.3°
*c* = 11.8745 (9) Å	µ = 0.09 mm^−^^1^
α = 63.230 (7)°	*T* = 170 K
β = 80.596 (6)°	Block, yellow
γ = 79.970 (6)°	0.28 × 0.25 × 0.24 mm
*V* = 1091.69 (14) Å^3^	

### Data collection

**Table:**

Oxford Diffraction Xcalibur Eos Gemini diffractometer	5644 independent reflections
Radiation source: Enhance (Mo) X-ray Source	4698 reflections with *I* > 2σ(*I*)
graphite	*R*_int_ = 0.012
Detector resolution: 16.1500 pixels mm^-1^	θ_max_ = 28.7°, θ_min_ = 3.3°
ω scans	*h* = −12→11
Absorption correction: multi-scan (*CrysAlis RED*; Oxford Diffraction, 2010)	*k* = −15→15
*T*_min_ = 0.976, *T*_max_ = 0.979	*l* = −15→16
10062 measured reflections	

### Refinement

**Table:**

Refinement on *F*^2^	Primary atom site location: structure-invariant direct methods
Least-squares matrix: full	Secondary atom site location: difference Fourier map
*R*[*F*^2^ > 2σ(*F*^2^)] = 0.053	Hydrogen site location: inferred from neighbouring sites
*wR*(*F*^2^) = 0.157	H atoms treated by a mixture of independent and constrained refinement
*S* = 1.05	*w* = 1/[σ^2^(*F*_o_^2^) + (0.0864*P*)^2^ + 0.2278*P*] where *P* = (*F*_o_^2^ + 2*F*_c_^2^)/3
5644 reflections	(Δ/σ)_max_ < 0.001
299 parameters	Δρ_max_ = 0.36 e Å^−^^3^
2 restraints	Δρ_min_ = −0.20 e Å^−^^3^

### Special details

**Table:**

Experimental. ^1^H NMR (600 MHz, DMSO-d6) δ 12.9 (bs, 1H), 7.85 7.84 (d, J = 8.0 Hz, 1H), 7.62 7.61 (d, J = 8.1 Hz, 1H), 7.42 7.39 (t, J = 7.4 Hz, 1H), 7.34–7.31 (t, J = 7.7 Hz, 1H), 1.44 (s, 6H). ^13^C NMR (150 MHz, DMSO-d_6_) δ 206.6, 175.2, 171.0, 140.0, 125.4, 123.4, 121.5, 121.1, 121.0, 113.6, 41.6, 22.9; IR ν(KBr) 3418, 1750, 1665, 1469, 1453, 1210, 1152, 1094, 1013; UV λ_max_ (95% MeOH) 256, 264, 272, 342 nm. Anal. Calcd for C_13_H_11_NO_2_: C, 73.22; H, 5.20; N 6.57. Found: C, 72.48; H, 5.30; N 6.40.
Geometry. All esds (except the esd in the dihedral angle between two l.s. planes) are estimated using the full covariance matrix. The cell esds are taken into account individually in the estimation of esds in distances, angles and torsion angles; correlations between esds in cell parameters are only used when they are defined by crystal symmetry. An approximate (isotropic) treatment of cell esds is used for estimating esds involving l.s. planes.
Refinement. Refinement of *F*^2^ against ALL reflections. The weighted *R*-factor *wR* and goodness of fit *S* are based on *F*^2^, conventional *R*-factors *R* are based on *F*, with *F* set to zero for negative *F*^2^. The threshold expression of *F*^2^ > σ(*F*^2^) is used only for calculating *R*-factors(gt) *etc*. and is not relevant to the choice of reflections for refinement. *R*-factors based on *F*^2^ are statistically about twice as large as those based on *F*, and *R*- factors based on ALL data will be even larger.

### Fractional atomic coordinates and isotropic or equivalent isotropic displacement parameters (Å^2^)

**Table:**

	*x*	*y*	*z*	*U*_iso_*/*U*_eq_	
O1	0.75420 (16)	0.86467 (16)	0.18664 (11)	0.0673 (4)	
O2	1.05510 (14)	0.75658 (14)	0.25613 (11)	0.0591 (3)	
N1	0.76832 (12)	0.67293 (11)	0.63752 (10)	0.0329 (2)	
H1NA	0.6811 (16)	0.6811 (16)	0.6806 (15)	0.039*	
C1	0.67431 (15)	0.78837 (13)	0.41344 (12)	0.0330 (3)	
C2	0.78552 (17)	0.80931 (15)	0.29437 (13)	0.0413 (3)	
C3	0.94630 (16)	0.75106 (15)	0.33304 (13)	0.0398 (3)	
C4	0.93046 (14)	0.69760 (13)	0.46734 (12)	0.0330 (3)	
C5	1.01358 (15)	0.62976 (14)	0.57632 (13)	0.0358 (3)	
C6	1.16300 (17)	0.58148 (19)	0.59556 (18)	0.0522 (4)	
H6A	1.2361	0.5907	0.5260	0.063*	
C7	1.2022 (2)	0.5200 (2)	0.7181 (2)	0.0653 (5)	
H7A	1.3038	0.4862	0.7326	0.078*	
C8	1.0970 (2)	0.5060 (2)	0.82122 (19)	0.0618 (5)	
H8A	1.1280	0.4626	0.9044	0.074*	
C9	0.94890 (19)	0.55398 (18)	0.80488 (15)	0.0496 (4)	
H9A	0.8771	0.5453	0.8751	0.060*	
C10	0.90872 (15)	0.61552 (14)	0.68187 (13)	0.0351 (3)	
C11	0.78265 (14)	0.71996 (12)	0.51124 (11)	0.0289 (3)	
C12	0.6013 (2)	0.92013 (15)	0.40795 (16)	0.0497 (4)	
H12D	0.5338	0.9061	0.4851	0.074*	
H12E	0.5442	0.9664	0.3338	0.074*	
H12F	0.6791	0.9724	0.4014	0.074*	
C13	0.55503 (18)	0.70394 (18)	0.42566 (17)	0.0495 (4)	
H13D	0.4878	0.6899	0.5029	0.074*	
H13E	0.6036	0.6194	0.4300	0.074*	
H13F	0.4972	0.7484	0.3519	0.074*	
N1A	0.10484 (12)	0.81468 (12)	1.00094 (10)	0.0343 (3)	
H1NB	0.0732 (19)	0.8074 (16)	1.0812 (13)	0.041*	
O1A	0.61629 (13)	0.64860 (15)	0.94255 (14)	0.0651 (4)	
O2A	0.46978 (13)	0.73188 (12)	0.71755 (11)	0.0529 (3)	
C1A	0.38431 (14)	0.72921 (13)	1.03441 (13)	0.0336 (3)	
C2A	0.48880 (15)	0.69922 (14)	0.93239 (15)	0.0390 (3)	
C3A	0.40683 (16)	0.74091 (13)	0.81363 (13)	0.0370 (3)	
C4A	0.25841 (14)	0.78537 (13)	0.84578 (12)	0.0320 (3)	
C5A	0.11410 (15)	0.83713 (13)	0.79856 (12)	0.0333 (3)	
C6A	0.05507 (19)	0.87076 (16)	0.68526 (14)	0.0452 (3)	
H6AA	0.1159	0.8600	0.6169	0.054*	
C7A	−0.0938 (2)	0.92010 (18)	0.67500 (16)	0.0538 (4)	
H7AA	−0.1353	0.9443	0.5980	0.065*	
C8A	−0.18450 (19)	0.93526 (19)	0.77470 (18)	0.0568 (4)	
H8AA	−0.2867	0.9692	0.7644	0.068*	
C9A	−0.12946 (17)	0.90208 (18)	0.88854 (16)	0.0490 (4)	
H9AA	−0.1916	0.9120	0.9568	0.059*	
C10A	0.02018 (15)	0.85378 (13)	0.89824 (12)	0.0345 (3)	
C11A	0.24447 (14)	0.77534 (12)	0.96840 (12)	0.0292 (3)	
C12A	0.37519 (19)	0.60627 (17)	1.15941 (16)	0.0512 (4)	
H12A	0.3434	0.5371	1.1456	0.077*	
H12B	0.3025	0.6255	1.2211	0.077*	
H12C	0.4738	0.5772	1.1919	0.077*	
C13A	0.43957 (19)	0.83897 (17)	1.04967 (18)	0.0518 (4)	
H13A	0.3713	0.8599	1.1125	0.078*	
H13B	0.4424	0.9168	0.9681	0.078*	
H13C	0.5403	0.8104	1.0781	0.078*	

### Atomic displacement parameters (Å^2^)

**Table:**

	*U*^11^	*U*^22^	*U*^33^	*U*^12^	*U*^13^	*U*^23^
O1	0.0641 (8)	0.0963 (10)	0.0291 (6)	0.0029 (7)	−0.0074 (5)	−0.0196 (6)
O2	0.0437 (6)	0.0938 (9)	0.0361 (6)	−0.0042 (6)	0.0117 (5)	−0.0315 (6)
N1	0.0271 (5)	0.0433 (6)	0.0241 (5)	−0.0003 (4)	0.0009 (4)	−0.0134 (4)
C1	0.0307 (6)	0.0383 (6)	0.0268 (6)	0.0014 (5)	−0.0042 (5)	−0.0127 (5)
C2	0.0429 (8)	0.0511 (8)	0.0273 (6)	−0.0021 (6)	−0.0012 (5)	−0.0165 (6)
C3	0.0350 (7)	0.0536 (8)	0.0310 (7)	−0.0033 (6)	0.0050 (5)	−0.0217 (6)
C4	0.0268 (6)	0.0428 (7)	0.0290 (6)	−0.0010 (5)	0.0014 (5)	−0.0175 (5)
C5	0.0284 (6)	0.0448 (7)	0.0356 (7)	0.0001 (5)	−0.0040 (5)	−0.0200 (6)
C6	0.0291 (7)	0.0757 (11)	0.0557 (10)	0.0045 (7)	−0.0059 (6)	−0.0351 (9)
C7	0.0378 (8)	0.0923 (14)	0.0728 (13)	0.0151 (9)	−0.0260 (9)	−0.0432 (11)
C8	0.0568 (11)	0.0806 (13)	0.0488 (10)	0.0086 (9)	−0.0269 (8)	−0.0273 (9)
C9	0.0471 (8)	0.0654 (10)	0.0334 (7)	0.0002 (7)	−0.0100 (6)	−0.0190 (7)
C10	0.0308 (6)	0.0429 (7)	0.0306 (6)	−0.0012 (5)	−0.0042 (5)	−0.0157 (5)
C11	0.0272 (6)	0.0335 (6)	0.0242 (6)	−0.0014 (4)	−0.0002 (4)	−0.0124 (5)
C12	0.0527 (9)	0.0409 (8)	0.0455 (8)	0.0076 (7)	−0.0038 (7)	−0.0149 (7)
C13	0.0394 (8)	0.0611 (9)	0.0523 (9)	−0.0075 (7)	−0.0085 (7)	−0.0263 (8)
N1A	0.0278 (5)	0.0469 (6)	0.0250 (5)	0.0028 (4)	−0.0006 (4)	−0.0159 (5)
O1A	0.0302 (6)	0.0908 (10)	0.0778 (9)	0.0113 (6)	−0.0046 (6)	−0.0463 (8)
O2A	0.0440 (6)	0.0689 (7)	0.0422 (6)	0.0004 (5)	0.0130 (5)	−0.0290 (6)
C1A	0.0280 (6)	0.0371 (6)	0.0338 (6)	0.0001 (5)	−0.0046 (5)	−0.0145 (5)
C2A	0.0272 (6)	0.0417 (7)	0.0461 (8)	−0.0024 (5)	0.0023 (5)	−0.0199 (6)
C3A	0.0342 (7)	0.0394 (7)	0.0345 (7)	−0.0039 (5)	0.0063 (5)	−0.0169 (5)
C4A	0.0311 (6)	0.0372 (6)	0.0257 (6)	−0.0032 (5)	0.0011 (5)	−0.0135 (5)
C5A	0.0327 (6)	0.0379 (6)	0.0271 (6)	−0.0041 (5)	−0.0013 (5)	−0.0126 (5)
C6A	0.0510 (9)	0.0542 (8)	0.0301 (7)	−0.0077 (7)	−0.0054 (6)	−0.0170 (6)
C7A	0.0543 (10)	0.0644 (10)	0.0390 (8)	−0.0065 (8)	−0.0201 (7)	−0.0142 (7)
C8A	0.0380 (8)	0.0717 (11)	0.0517 (10)	0.0019 (7)	−0.0157 (7)	−0.0179 (8)
C9A	0.0313 (7)	0.0662 (10)	0.0415 (8)	0.0043 (7)	−0.0037 (6)	−0.0199 (7)
C10A	0.0309 (6)	0.0406 (7)	0.0273 (6)	−0.0015 (5)	−0.0031 (5)	−0.0115 (5)
C11A	0.0266 (6)	0.0316 (6)	0.0261 (6)	−0.0010 (4)	0.0000 (4)	−0.0112 (5)
C12A	0.0402 (8)	0.0522 (9)	0.0417 (8)	0.0038 (6)	−0.0067 (6)	−0.0054 (7)
C13A	0.0453 (9)	0.0570 (9)	0.0638 (11)	−0.0044 (7)	−0.0134 (8)	−0.0336 (8)

### Geometric parameters (Å, °)

**Table:**

O1—C2	1.2023 (18)	N1A—C11A	1.3301 (16)
O2—C3	1.2224 (17)	N1A—C10A	1.4057 (17)
N1—C11	1.3371 (16)	N1A—H1NB	0.919 (14)
N1—C10	1.4009 (17)	O1A—C2A	1.2042 (17)
N1—H1NA	0.887 (14)	O2A—C3A	1.2304 (17)
C1—C11	1.4910 (17)	C1A—C11A	1.4949 (17)
C1—C12	1.5260 (19)	C1A—C12A	1.525 (2)
C1—C13	1.530 (2)	C1A—C13A	1.532 (2)
C1—C2	1.5470 (19)	C1A—C2A	1.5424 (19)
C2—C3	1.551 (2)	C2A—C3A	1.545 (2)
C3—C4	1.4192 (19)	C3A—C4A	1.4151 (18)
C4—C11	1.3863 (17)	C4A—C11A	1.3937 (17)
C4—C5	1.4359 (19)	C4A—C5A	1.4370 (18)
C5—C6	1.3920 (19)	C5A—C6A	1.3939 (19)
C5—C10	1.4100 (19)	C5A—C10A	1.4095 (18)
C6—C7	1.379 (3)	C6A—C7A	1.378 (2)
C6—H6A	0.9500	C6A—H6AA	0.9500
C7—C8	1.392 (3)	C7A—C8A	1.390 (3)
C7—H7A	0.9500	C7A—H7AA	0.9500
C8—C9	1.376 (2)	C8A—C9A	1.383 (2)
C8—H8A	0.9500	C8A—H8AA	0.9500
C9—C10	1.386 (2)	C9A—C10A	1.3822 (19)
C9—H9A	0.9500	C9A—H9AA	0.9500
C12—H12D	0.9800	C12A—H12A	0.9800
C12—H12E	0.9800	C12A—H12B	0.9800
C12—H12F	0.9800	C12A—H12C	0.9800
C13—H13D	0.9800	C13A—H13A	0.9800
C13—H13E	0.9800	C13A—H13B	0.9800
C13—H13F	0.9800	C13A—H13C	0.9800
			
C11—N1—C10	108.76 (11)	C11A—N1A—C10A	108.31 (11)
C11—N1—H1NA	121.8 (11)	C11A—N1A—H1NB	122.5 (11)
C10—N1—H1NA	129.4 (11)	C10A—N1A—H1NB	129.0 (11)
C11—C1—C12	113.21 (12)	C11A—C1A—C12A	114.55 (12)
C11—C1—C13	113.17 (12)	C11A—C1A—C13A	111.77 (11)
C12—C1—C13	110.52 (13)	C12A—C1A—C13A	111.60 (14)
C11—C1—C2	98.41 (10)	C11A—C1A—C2A	97.98 (10)
C12—C1—C2	109.97 (12)	C12A—C1A—C2A	110.59 (12)
C13—C1—C2	110.99 (12)	C13A—C1A—C2A	109.51 (12)
O1—C2—C1	125.61 (14)	O1A—C2A—C1A	125.67 (14)
O1—C2—C3	124.13 (14)	O1A—C2A—C3A	123.63 (14)
C1—C2—C3	110.25 (11)	C1A—C2A—C3A	110.69 (11)
O2—C3—C4	132.30 (14)	O2A—C3A—C4A	132.76 (14)
O2—C3—C2	123.08 (13)	O2A—C3A—C2A	122.40 (13)
C4—C3—C2	104.62 (11)	C4A—C3A—C2A	104.84 (11)
C11—C4—C3	110.28 (12)	C11A—C4A—C3A	109.89 (12)
C11—C4—C5	107.02 (11)	C11A—C4A—C5A	107.00 (11)
C3—C4—C5	142.68 (12)	C3A—C4A—C5A	143.11 (13)
C6—C5—C10	119.17 (14)	C6A—C5A—C10A	119.22 (13)
C6—C5—C4	135.02 (13)	C6A—C5A—C4A	135.42 (13)
C10—C5—C4	105.80 (11)	C10A—C5A—C4A	105.36 (11)
C7—C6—C5	118.31 (16)	C7A—C6A—C5A	118.32 (15)
C7—C6—H6A	120.8	C7A—C6A—H6AA	120.8
C5—C6—H6A	120.8	C5A—C6A—H6AA	120.8
C6—C7—C8	121.72 (16)	C6A—C7A—C8A	121.50 (14)
C6—C7—H7A	119.1	C6A—C7A—H7AA	119.3
C8—C7—H7A	119.1	C8A—C7A—H7AA	119.3
C9—C8—C7	121.20 (16)	C9A—C8A—C7A	121.55 (15)
C9—C8—H8A	119.4	C9A—C8A—H8AA	119.2
C7—C8—H8A	119.4	C7A—C8A—H8AA	119.2
C8—C9—C10	117.32 (16)	C10A—C9A—C8A	116.88 (15)
C8—C9—H9A	121.3	C10A—C9A—H9AA	121.6
C10—C9—H9A	121.3	C8A—C9A—H9AA	121.6
C9—C10—N1	129.67 (13)	C9A—C10A—N1A	128.81 (13)
C9—C10—C5	122.27 (13)	C9A—C10A—C5A	122.53 (13)
N1—C10—C5	108.06 (11)	N1A—C10A—C5A	108.66 (11)
N1—C11—C4	110.35 (11)	N1A—C11A—C4A	110.66 (11)
N1—C11—C1	133.24 (11)	N1A—C11A—C1A	132.96 (12)
C4—C11—C1	116.41 (11)	C4A—C11A—C1A	116.33 (11)
C1—C12—H12D	109.5	C1A—C12A—H12A	109.5
C1—C12—H12E	109.5	C1A—C12A—H12B	109.5
H12D—C12—H12E	109.5	H12A—C12A—H12B	109.5
C1—C12—H12F	109.5	C1A—C12A—H12C	109.5
H12D—C12—H12F	109.5	H12A—C12A—H12C	109.5
H12E—C12—H12F	109.5	H12B—C12A—H12C	109.5
C1—C13—H13D	109.5	C1A—C13A—H13A	109.5
C1—C13—H13E	109.5	C1A—C13A—H13B	109.5
H13D—C13—H13E	109.5	H13A—C13A—H13B	109.5
C1—C13—H13F	109.5	C1A—C13A—H13C	109.5
H13D—C13—H13F	109.5	H13A—C13A—H13C	109.5
H13E—C13—H13F	109.5	H13B—C13A—H13C	109.5
			
C11—C1—C2—O1	−178.28 (17)	C11A—C1A—C2A—O1A	−174.04 (15)
C12—C1—C2—O1	−59.7 (2)	C12A—C1A—C2A—O1A	−54.0 (2)
C13—C1—C2—O1	62.9 (2)	C13A—C1A—C2A—O1A	69.41 (19)
C11—C1—C2—C3	0.43 (15)	C11A—C1A—C2A—C3A	4.93 (13)
C12—C1—C2—C3	118.96 (14)	C12A—C1A—C2A—C3A	124.99 (13)
C13—C1—C2—C3	−118.44 (13)	C13A—C1A—C2A—C3A	−111.62 (13)
O1—C2—C3—O2	−0.2 (3)	O1A—C2A—C3A—O2A	−4.3 (2)
C1—C2—C3—O2	−178.95 (15)	C1A—C2A—C3A—O2A	176.71 (13)
O1—C2—C3—C4	179.30 (17)	O1A—C2A—C3A—C4A	175.40 (15)
C1—C2—C3—C4	0.57 (16)	C1A—C2A—C3A—C4A	−3.60 (15)
O2—C3—C4—C11	178.02 (17)	O2A—C3A—C4A—C11A	−179.98 (16)
C2—C3—C4—C11	−1.44 (16)	C2A—C3A—C4A—C11A	0.37 (15)
O2—C3—C4—C5	−0.2 (3)	O2A—C3A—C4A—C5A	0.6 (3)
C2—C3—C4—C5	−179.69 (18)	C2A—C3A—C4A—C5A	−179.08 (17)
C11—C4—C5—C6	−179.60 (17)	C11A—C4A—C5A—C6A	179.17 (16)
C3—C4—C5—C6	−1.3 (3)	C3A—C4A—C5A—C6A	−1.4 (3)
C11—C4—C5—C10	−0.31 (15)	C11A—C4A—C5A—C10A	−0.72 (15)
C3—C4—C5—C10	177.97 (19)	C3A—C4A—C5A—C10A	178.74 (17)
C10—C5—C6—C7	0.7 (3)	C10A—C5A—C6A—C7A	0.4 (2)
C4—C5—C6—C7	179.91 (18)	C4A—C5A—C6A—C7A	−179.47 (16)
C5—C6—C7—C8	−0.3 (3)	C5A—C6A—C7A—C8A	−0.6 (3)
C6—C7—C8—C9	−0.4 (3)	C6A—C7A—C8A—C9A	0.3 (3)
C7—C8—C9—C10	0.6 (3)	C7A—C8A—C9A—C10A	0.3 (3)
C8—C9—C10—N1	−179.92 (16)	C8A—C9A—C10A—N1A	178.99 (15)
C8—C9—C10—C5	−0.2 (3)	C8A—C9A—C10A—C5A	−0.6 (3)
C11—N1—C10—C9	−179.70 (16)	C11A—N1A—C10A—C9A	−179.67 (15)
C11—N1—C10—C5	0.51 (16)	C11A—N1A—C10A—C5A	−0.06 (16)
C6—C5—C10—C9	−0.5 (2)	C6A—C5A—C10A—C9A	0.2 (2)
C4—C5—C10—C9	−179.92 (15)	C4A—C5A—C10A—C9A	−179.88 (14)
C6—C5—C10—N1	179.31 (14)	C6A—C5A—C10A—N1A	−179.42 (13)
C4—C5—C10—N1	−0.11 (15)	C4A—C5A—C10A—N1A	0.48 (15)
C10—N1—C11—C4	−0.73 (15)	C10A—N1A—C11A—C4A	−0.41 (15)
C10—N1—C11—C1	179.04 (14)	C10A—N1A—C11A—C1A	176.95 (13)
C3—C4—C11—N1	−178.24 (12)	C3A—C4A—C11A—N1A	−178.94 (11)
C5—C4—C11—N1	0.65 (16)	C5A—C4A—C11A—N1A	0.72 (15)
C3—C4—C11—C1	1.95 (17)	C3A—C4A—C11A—C1A	3.22 (16)
C5—C4—C11—C1	−179.16 (11)	C5A—C4A—C11A—C1A	−177.13 (11)
C12—C1—C11—N1	62.8 (2)	C12A—C1A—C11A—N1A	60.7 (2)
C13—C1—C11—N1	−63.97 (19)	C13A—C1A—C11A—N1A	−67.49 (19)
C2—C1—C11—N1	178.83 (15)	C2A—C1A—C11A—N1A	177.73 (14)
C12—C1—C11—C4	−117.46 (14)	C12A—C1A—C11A—C4A	−122.05 (14)
C13—C1—C11—C4	115.79 (14)	C13A—C1A—C11A—C4A	109.76 (14)
C2—C1—C11—C4	−1.41 (15)	C2A—C1A—C11A—C4A	−5.02 (14)

### Hydrogen-bond geometry (Å, °)

**Table:**

*D*—H···*A*	*D*—H	H···*A*	*D*···*A*	*D*—H···*A*
N1—H1NA···O2A	0.89 (1)	1.95 (1)	2.8025 (16)	161.(2)
N1A—H1NB···O2^i^	0.92 (1)	1.87 (1)	2.7686 (16)	164.(2)
C9A—H9AA···O1^i^	0.95	2.51	3.376 (2)	152.
C12A—H12B···O2^i^	0.98	2.57	3.430 (2)	146.

Symmetry codes: (i) *x*−1, *y*, *z*+1.

## References

[bb1] Allen, F. H., Kennard, O., Watson, D. G., Brammer, L., Orpen, A. G. & Taylor, R. (1987). *J. Chem. Soc. Perkin Trans. 2*, pp. S1–19.

[bb2] Belsey, S., Danks, T. N. & Wagner, G. (2006). *Synth. Commun.* **36**, 1019–1024.

[bb3] Bergman, J. & Venemalm, L. (1992). *Tetrahedron* **48**, 759–768.

[bb4] Cheng, K.-F., Chan, K.-P. & Lai, T.-F. (1991). *J. Chem. Soc. Perkin Trans. 1*, pp. 2461–2465.

[bb5] Cheng, K.-F. & Cheung, M.-K. (1996). *J. Chem. Soc., Perkin Trans. 1*, pp. 1213–1218.

[bb6] Churruca, F., Fousteris, M., Ishikawa, Y., von Wantoch Rekowski, M., Hounsou, C., Surrey, T. & Giannis, A. (2010). *Org. Lett.* **12**, 2096–2099. 10.1021/ol100579w 20387880

[bb7] Ekebergh, A., Karlsson, I., Mete, R., Pan, Y., Börje, A. & Mårtensson, J. (2011). *Org. Lett.* **13**, 4458–4461. 10.1021/ol201812n PMC3164230 21786790

[bb8] Garcia-Pichel, F. & Castenholz, R. W. (1991). *J. Phycol.* **27**, 395–409.

[bb9] Garcia-Pichel, F., Sherry, N. D. & Castenholz, R. W. (1992). *Photochem. Photobiol.* **56**, 17–23. 10.1111/j.1751-1097.1992.tb09596.x 1508978

[bb10] Gribble, G. W., Barden, T. C. & Johnson, D. A. (1988). *Tetrahedron*, **44**, 3195–3202.

[bb11] Ishikura, M., Imaizumi, K. & Katagiri, N. (2000). *Heterocycles*, **53**, 2201–2220.

[bb12] Jacquemard, U., Bénéteau, V., Lefoix, M., Routier, S., Mérour, J.-Y. & Coudert, G. (2004). *Tetrahedron*, **60**, 10039–10047.

[bb13] Jordan, J. A., Gribble, G. W. & Badenock, J. C. (2011). *Tetrahedron Lett.* **52**, 6772–6774.

[bb14] Kobayashi, A., Kajiyama, S.-I., Inawaka, K., Kanzaki, H. & Kawazu, K. Z. (1994). *Z. Naturforsch. Teil C*, **49**, 464–470.

[bb15] Miki, Y., Hachiken, H., Kawazoe, A., Tsuzaki, Y. & Yanase, N. (2001). *Heterocycles*, **55**, 1291–1299.

[bb16] Ouyang, Y., Koike, K. & Ohmoto, T. (1994). *Phytochemistry*, **37**, 575–578. 10.1016/s0031-9422(00)89758-7 7765437

[bb17] Oxford Diffraction (2010). *CrysAlis PRO* and *CrysAlis RED* Oxford Diffraction Ltd, Yarnton, England.

[bb18] Ploutno, A. & Carmeli, S. (2001). *J. Nat. Prod.* **64**, 544–545. 10.1021/np000562w 11325247

[bb19] Proteau, P. J., Gerwick, W. H., Garcia-Pichel, F. & Castenholz, R. (1993). *Cell. Mol. Life Sci.* **49**, 825–829. 10.1007/BF01923559 8405307

[bb20] Sheldrick, G. M. (2008). *Acta Cryst.* A**64**, 112–122. 10.1107/S0108767307043930 18156677

[bb21] Song, C., Knight, D. W. & Whatton, M. A. (2006). *Org. Lett.* **8**, 163–166. 10.1021/ol052683z 16381593

[bb22] Xu, P.-F., Chen, Y.-S., Lin, S.-I. & Lu, T.-J. (2002). *J. Org. Chem.* **67**, 2309–2314. 10.1021/jo011139a 11925246

